# Structural Impacts of Drug-Resistance Mutations Appearing in HIV-2 Protease

**DOI:** 10.3390/molecules26030611

**Published:** 2021-01-25

**Authors:** Pierre Laville, Michel Petitjean, Leslie Regad

**Affiliations:** Université de Paris, BFA, UMR 8251, CNRS, ERL U1133, Inserm, F-75013 Paris, France; pierre.laville@u-paris.fr (P.L.); petitjean.chiral@gmail.com (M.P.)

**Keywords:** drug-resistance mutations, HIV-2 protease, structural characterization, induced structural deformations

## Abstract

The use of antiretroviral drugs is accompanied by the emergence of HIV-2 resistances. Thus, it is important to elucidate the mechanisms of resistance to antiretroviral drugs. Here, we propose a structural analysis of 31 drug-resistant mutants of HIV-2 protease (PR2) that is an important target against HIV-2 infection. First, we modeled the structures of each mutant. We then located structural shifts putatively induced by mutations. Finally, we compared wild-type and mutant inhibitor-binding pockets and interfaces to explore the impacts of these induced structural deformations on these two regions. Our results showed that one mutation could induce large structural rearrangements in side-chain and backbone atoms of mutated residue, in its vicinity or further. Structural deformations observed in side-chain atoms are frequent and of greater magnitude, that confirms that to fight drug resistance, interactions with backbone atoms should be favored. We showed that these observed structural deformations modify the conformation, volume, and hydrophobicity of the binding pocket and the composition and size of the PR2 interface. These results suggest that resistance mutations could alter ligand binding by modifying pocket properties and PR2 stability by impacting its interface. Our results reinforce the understanding of the effects of mutations that occurred in PR2 and the different mechanisms of PR2 resistance.

## 1. Introduction

The human immunodeficiency virus (HIV) affects humans and causes the acquired immunodeficiency syndrome (AIDS). The treatment against HIV-1 infection corresponds to the same molecules that target four HIV proteins: the fusion protein, protease (PR), integrase, and reverse transcriptase. The same molecules are used to fight HIV-2 infection but HIV-2 is naturally resistant to most of these inhibitors [1,2,3,4,5,6,7,8,9]. Thus, it is important to develop new molecules that inhibit the HIV-2 replication, particularly against the HIV-2 protease (PR2).

PR is a homodimer that plays a major role in the virus maturation process: it hydrolyzes the viral polyproteins Gag and Gag-Pol, causing the development of immature virions. There are currently nine protease inhibitors (PIs) clinically recommended for treating HIV-1 infection [10]. These drugs bind to the PR catalytic site in the interface of the two monomers. This binding induces structural changes in the entire PR2, particularly in the flap region allowing the closing of binding pocket [10,11,12]. PR2 is naturally resistant to most of these PIs and only three of them are recommended for the treatment of HIV-2 infection: darunavir (DRV), saquinavir (SQV), and lopinavir (LPV) [1,10]. The natural resistance of PR2 is explained by amino-acid changes between PR1 and PR2 that induce structural changes in the entire structure [13]. Some of these structural changes, located in the binding pocket, modify properties and conformation of the PI-binding pocket and the internal interactions between PR2 and PIs [3,5,14,15,16,17,18,19,20]. Other structural changes, occurring in the elbow and flap regions, alter the transition between the open and closed forms involved in ligand binding [13,21,22].

In addition to its natural resistance, many acquired resistance mutations appear in PR2. The identification of these mutations have been performed using genome sequencing studies of HIV-2 virus extracted from infected patients [2,4,5,6,8,23,24,25,26,27,28]. For example, it has been shown that the V47A, I50V, I54M, I82F, I84V, and L90M mutations lead to several PIs resistance [2,4,5,7,8]. Phenotypic susceptibility assays were used to confirm the resistance of some mutants [2,4,5,8,26,29]. For example, genotypic and phenotypic analyses showed that the I82F mutation causes resistance to indinavir (IDV) [26]. Furthermore, this mutation has been identified as causing hypersusceptibility to both DRV and SQV using phenotypic assays [2]. In addition, combinations of several mutations confer high resistance to several PIs [2,4,5,7,8,30]. For example, the I54M and I82F mutations induce resistance to all PIs and the V62A/L99F mutant is resistant to nelfinavir, IDV, and LPV [26]. Few studies have focused on the structural analysis of the impacts of drug-resistance mutations reported in PR2 because no tridimensional (3D) structure of PR2 mutant have been solved. These structural studies could help to understand the atomistic mechanism of resistance mutations. In our previous work, we performed the first structural analysis of the impact of 30 drug-resistant mutants of PR2 based on modeled structures. More precisely, we explored the consequences of drug-resistance mutations on PR2 structural asymmetry, an important property for ligand-binding and the target deformation [31]. Our findings suggested three possible resistance mechanisms of PR2: (i) mutations that induce structural changes in the binding pocket that could directly alter PI-binding, (ii) mutations that could impact the properties and conformation of the binding pocket by inducing structural changes in residues outside of the binding pocket but involved in interaction with pocket residues, and (iii) mutations that could modify the PR2 interface and its stability through structural changes in interface residues. These results have been based on PR2 backbone analysis. However, a better characterization of the structural impacts of drug-resistance mutations on PR2 structure including side-chain atoms could help in a better understanding of different proposed mechanisms.

In this study, we structurally analyzed a set of 31 drug-resistant mutants of PR2 that was updated relative to [31]. The 3D structure of each mutant was modeled and its structure was compared to the wild-type PR2 to locate structural rearrangements induced by drug-resistance mutations at backbone and side-chain atoms. The study reported that drug-resistance mutations could impact the flexbility of PR2 and the closing binding pocket, conformation and properties of PI-pocket and the composition and size of the PR2 interface.

## 2. Results

**Figure 1:**
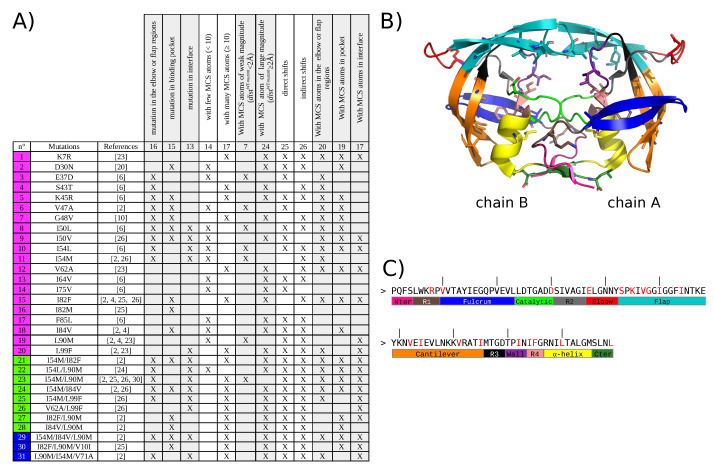
Description of the 31 drug-resistant mutants studied in this analysis. (**A**) Table listing the 31 drug-resistant mutants studied in this analysis. Single mutants are colored in magenta, double mutants in green and triple mutants in blue. (**B**) Location on PR2 structure of the 22 drug-resistance mutations included in the 31 mutant set. PR2 is represented in cartoon mode and colored according to the 13 regions defined in [34,35]. Mutations are represented in stick mode. (**C**) Amino acid sequence of PR2 presenting the limit of the 13 PR2 regions. All the mutated residues are colored in red in the sequence.

**Figure A1:**
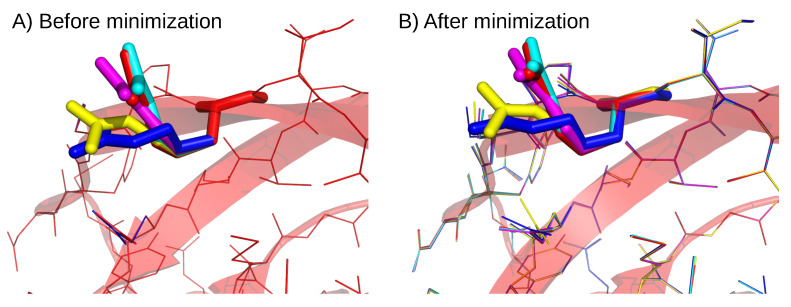
Illustration of the different rotamers for the arginine in position 45. Superimposition of the five structures of the K45R mutant before (**A**) and after the minimization process (**B**). Mutant structures are presented in line and cartoon mode. Residues R45 are presented in sticks.

We studied the impact of 22 drug-resistance mutations on PR2 structure. These mutations appeared alone or in combination with others (two or three mutations per mutant) resulting in a set of 31 mutants, Figure 1A. First, mutant structures were modeled using FoldX software [32] and an energetic minimization step using the crystallographic structure of the wild-type PR2 (PDB code: 3EBZ [33]). Five 3D structures were built for each mutant to consider the different possible rotamers per amino acids as illustrated by Figure A1. This mutagenesis process resulted in a set of 155 mutant structures. The crystallographic structure of the wild-type PR2 (PDB code: 3EBZ [33]) was also energetically minimized with the protocol used for mutant structures. In the following, the minimized wild-type structure was referred as the wild-type structure and its structure was compared to the minimized mutant structures.

### 2.1. Identification of Atom Shifts in the Mutant-Structure Set

**Figure 2:**
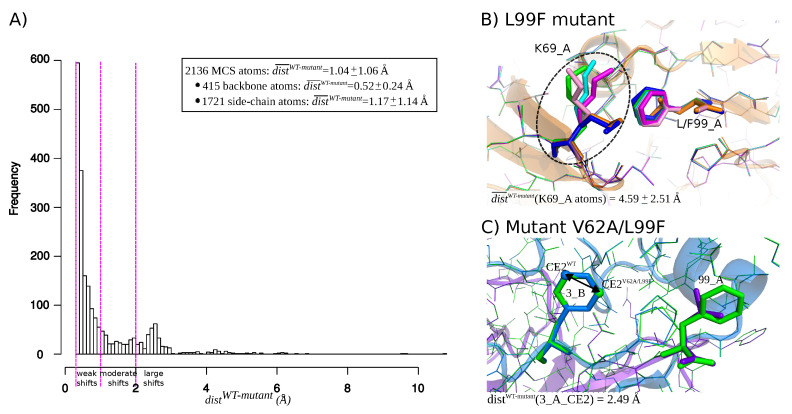
(**A**) Distribution of $dist^{WT-mutant}$ distances for MCS atoms extracted from the set of 155 mutant structures. Magenta lines corresponds to the cutoffs used to define a weak shift (0.3 Å < $dist^{WT-mutant}$ < 1 Å), moderate shifts (1 Å $\leq dist^{WT-mutant}$ < 2 Å), and large shifts ($dist^{WT-mutant}$ > 2 Å). (**B**,**C**) Illustration of atom shifts in the L99F (**A**) and V62A/L99F (**B**) mutants. (**B**) Superimposition of the five structures of the L99F mutant and the wild-type structure. Wild-type structure is colored in orange and represented in line and cartoon modes. The five structure of the mutant are represented in line mode and colored in magenta, cyan, blue, green, and pink. The L99F mutated residue is represented in stick mode. (**C**) Illustration of structural shift occuring at residue 3_B in the V62A/L99F mutant. The wild-type structure is represented in cartoon and line modes and colored according to its two chains: chain A is colored in purple and chain B is colored in marien blue. The mutant V62A/L99F structure is represented in lines and colored in green. The mutated residue 99_A and shifted residue 3_A are represented in stick mode. The arrow represents the $dist^{WT-mutant}$ of the CE atom of residue 3_B computed between the wild-type and the first structure of mutant V62A/L99F. $CE2^{V62A/L99F}$ and $CE2^{WT}$ correspond to atom CE of residue 3 of chain B in the V62A/L99F and wild-type structures and are represented in sphere mode.

**Figure A2:**
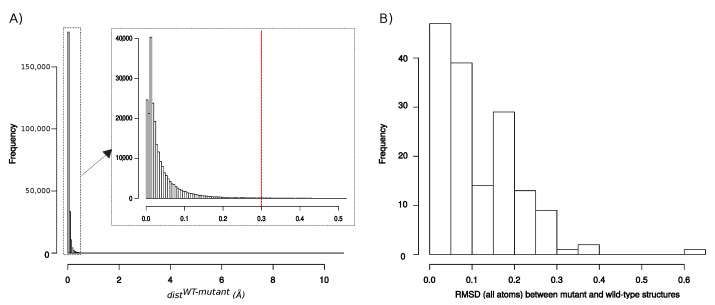
(**A**) Distribution of $dist^{WT-mutant}$ distances for each atom (hydrogen atoms were removed) in the set of 155 mutant structures. Red line corresponds the distance cutoff (0.3 Å) used to define an atom as shifted atom. (**B**) Distribution of RMSD (in Å) computed between wild-type and mutant structures. RMSD were computed using all atoms.

We first explored the structural deformations induced by mutations by locating shifted atoms in mutant structures relative to the wild-type structure. The shift of an atom was quantified by $dist^{WT-mutant}$, i.e., the distance between its positions in the wild-type and mutant structures. Figure A2 presents the distribution of $dist^{WT-mutant}$ of all atoms in the mutant structures set, except hydrogen atoms. These distances varied from 0.05 to 0.05, with 95% of atoms exhibited a $dist^{WT-mutant}$ smaller than 0.14 Å. These distances were summarized per structures by computing the RMSD between the wild-type and mutant structures (Figure A2). As expected, mutant structures exhibited close conformation than the wild-type, resulting in an average RMSD of 0.12 ± 0.09 Å. Only one mutant structure (one structure of the K7R mutant) exhibited a RMSD higher than 0.5 Å. Like in Liu et al., 2008 [36], we considered that a shift was significant if it had a magnitude higher than 0.3 Å because of uncertainties in the X-ray and mutant structures. Thus, to identify atom shifts induced by mutations, we retained the 2136 atoms with a $dist^{WT-mutant}$ higher than 0.3 Å in at least three structures of a given mutant. These selected atoms were denoted as “mutant-conserved shifted atoms” (MCS atoms) and the distribution of their $dist^{WT-mutant}$ is provided by Figure 2A. Most MCS atoms (70%) exhibited a shift of moderate magnitude (<1 Å). However, 17% of MCS atoms are of large magnitude (>2 Å), such as atom shifts detected in residue K69_A (Lysine 69 in chain A) in the L99F mutant that is illustrated in Figure 2B. Figure 2C presents another type of atom shift that corresponds to a flip of a ring of residue 3_B (residue 3 in chain B) in the five structures of the V62A/L99F mutant. This rearrangement type does not induce structural deformation and thus it does not seem to be linked to PI resistance.

As expected, side-chain atoms were overrepresented in the MCS atom set (Pearson’s Chi-squared Test *p*-value = $3\times 10^{-28}$) and they had larger $dist^{WT-mutant}$ value than backbone atoms (Student’s *t*-test *p*-value of $6\times 10^{-95}$). This means that drug-resistance mutations have more impacts on side-chain atoms than on backbone atoms. From the 2136 MCS atoms, 543 (=25%) are atoms of mutated residues. The shift of these atoms, named direct shifts, was a direct consequence of mutations. In contrast, 1593 (=75%) MCS atoms corresponded to indirect shifts, i.e., they occurred in non mutated residues, and their shifts resulted either from the intrinsic flexibility of atoms or from indirect impacts induced by the mutation through contacts between these atoms and mutated residues. Direct shifts had larger magnitude than indirect shifts, i.e., they exhibited an average higher $dist^{WT-mutant}$ distances than indirect shifts (Student’s *t*-test *p*-value = $3\times 10^{-13}$). To distinguish structural shifts resulting from mutation from those induced by flexibility, we detected MCS atoms in non minimized structures, i.e., mutant structures corresponded to the output of FoldX software. From this set of non-minimized structures, we located 883 MCS atoms, with 646 that were also detected as MCS atoms in the minimized structures set. This means that the shift of these MCS atoms observed in minimized structures (30% of MCS atoms) were the consequence of the mutation. As expected, these shifts, observed in minimized structures, occurred only on side-chain atoms and in or close to the mutated residue because FoldX software optimizes the configuration of only side chains in the vicinity of the mutated residue. The remained detected shifts were explained by the mutation and intrinsic flexibility of atoms.

**Figure 3:**
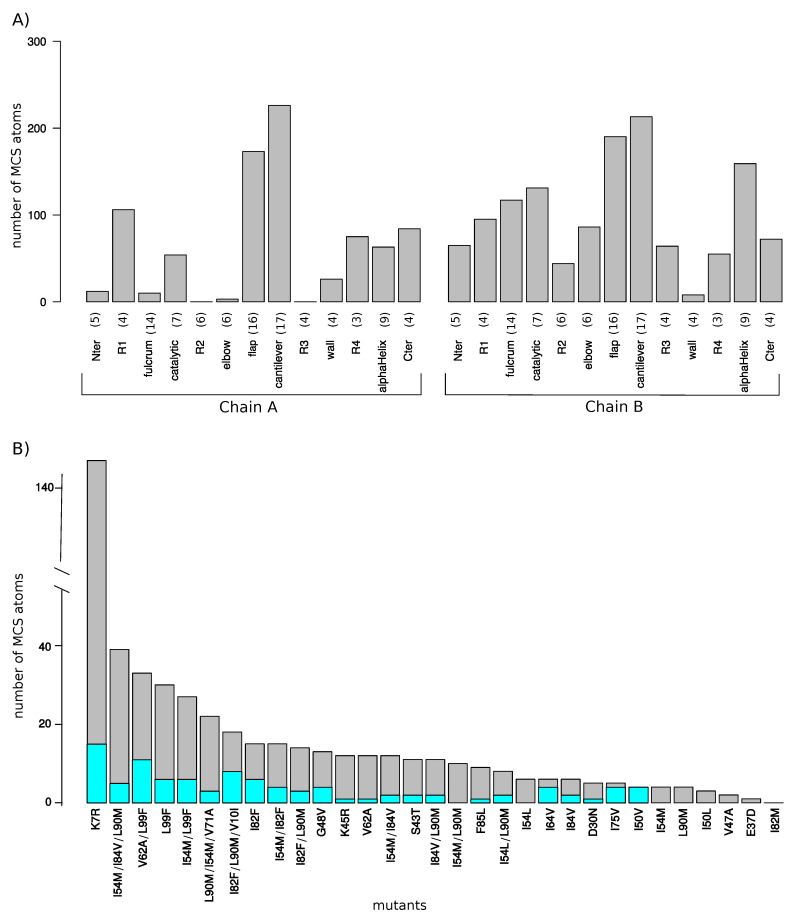
(**A**) Number of MCS atoms per mutant counted in the two PR2 chains. Each PR2 chain was divided in 13 regions according to [13]. Numbers in brackets indicate the size of each region in terms of amino acids. (**B**) Number of MCS atoms (grey) and MCS atoms with large magnitude ($dist^{WT-mutant}\geq 2$ Å, cyan) per mutants. Mutants were sorted according to their number of MCS atoms.

Figure 3A shows that the entire PR2 structure was sampled by MCS atoms. A total of eight regions had few MCS atoms, i.e., less than 50 MCS atoms were detected in the 155 mutant structures (Figure 3). In contrast, some regions exhibited many MCS atoms, such as the cantilever and flap regions of the two chains and the α-helix region of chain B, with more than 150 MCS atoms. In addition, an assymetry between the two chains in terms of number of MCS atoms was observed. Indeed, chain B contained more MCS atoms than chain A (*p*-value of the Pearson’s Chi-square test is of $5\times 10^{-24}$). For example, the Nter, fulcrum, elbow, and R3 regions of chain A present few structural shifts, while they exhibited lots of deformations in chain B. Thus, even though mutations occurred in the two chains, they did not impact in the same way the two monomers.

**Figure 4:**
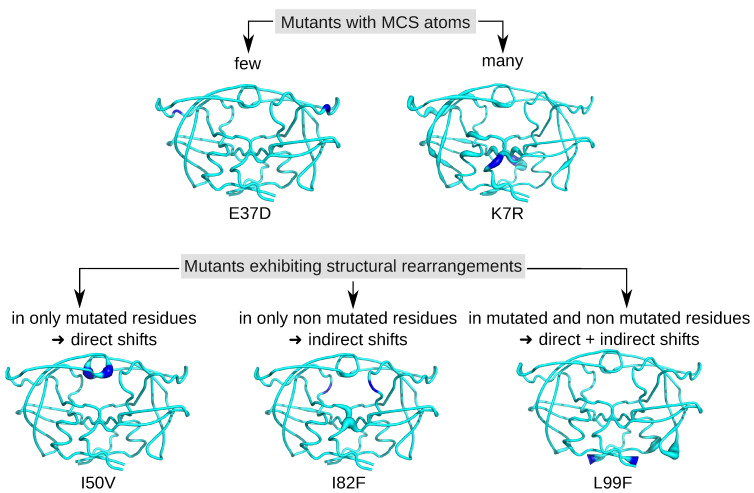
Location of the MCS atoms found in some mutants. PR2 is colored in cyan and represented in putty mode. The putty radius is relative to deformations induced by mutations: the higher the radius, the stronger the mutation-induced rearrangement. Mutated residues are colored in blue.

**Figure A3:**
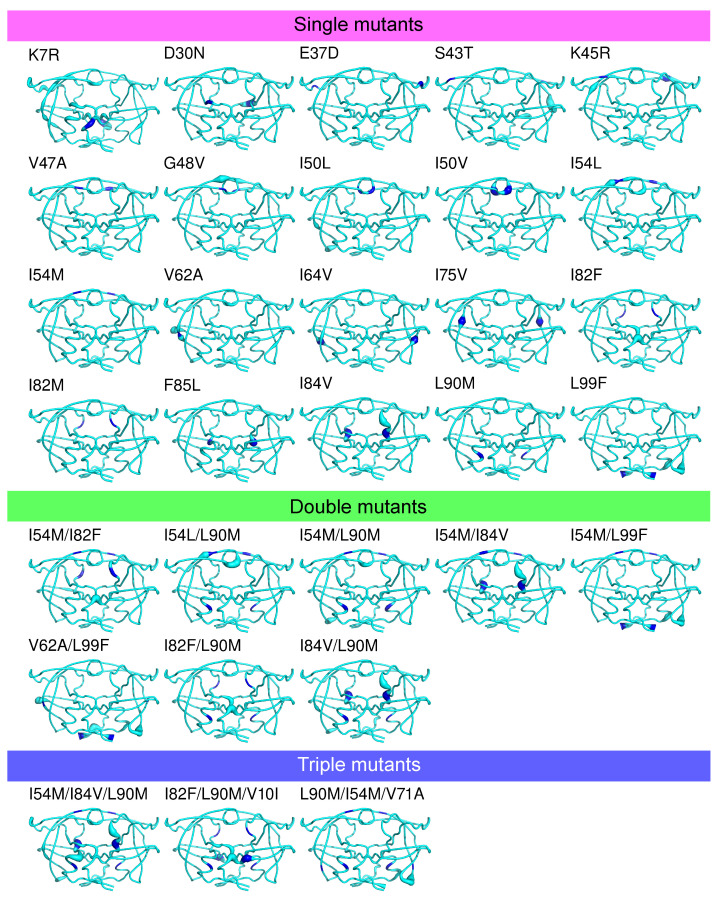
Location of the MCS atoms found in each mutant. PR2 is colored in cyan and represented in putty mode. The putty radius is relative to the deformation induced by mutations: the higher the radius, the stronger the mutation-induced rearrangement. Mutated residues are colored in blue.

Figure 3B presents the number of MCS atoms per mutants. On average, a mutant contained a moderate number of MCS atoms (16.26 ± 26.09). The I82M mutant was particular because it had no MCS atoms, revealing that the I82M mutation induced few structural changes in PR2 structure. In contrast, the K7R mutant was the mutant with the most MCS atoms (147 MCS located in 53 different residues as illustrated in Figure 4. A total of 14 mutants had less than 10 MCS atoms, indicating that these mutations induced few impacts on PR2 structure (Figure 1A). Except the I54L/L90M, all these mutants were single mutants. Although these mutants exhibited few deformations, 57% of them had shifts with a large magnitude ($dist^{WT-mutant}\geq 2$ Å). For example, the D30N mutant induced 5 MCS atoms with one exhibiting large shift of 2.9 Å, while the L90M mutant caused four small shifts at residues 90_A/B and 97_B with a magnitude varying from 0.35 to 0.48 Å (Figure 3B and Figure A3). In contrast, 17 mutants had many MCS atoms and all of these shifts had of large magnitude (Figure 3B). From these mutants, seven were single mutants, revealing that only one mutation could cause large deformations, such as those observed for the K7R and L99F mutants (Figure 4 and Figure A3).

According to the location of MCS atoms, we differentiated three types of mutations (Figure 4). The first mutations corresponded to four mutations that induced structural rearrangements only in the mutated residues, i.e., having only direct impacts. For example, the I50V mutation caused structural changes in two atoms of residue 50 of the two chains, a residue involved in the binding pocket, the dimer interface and the flap region (Figure 4). The second type of mutations grouped five mutations impacting residues in their vicinity or further in structure, i.e., inducing indirect structural changes, such as the I82F mutations (Figure 1). Indeed, the I82F mutation induced many atoms rearrangements in five non mutated residues (8_A, 8_B, 21_B, 27_B, 49_B) with some of them of large magnitude. In this mutant structure, residue 8_A is located in the vicinity of mutated residue 82_A (located at less than 5.5 Å), while residues 27_B and 49_B that are located at more than 6 Å of the mutated residue 82_A (Figure A8). The last type of mutations corresponded to mutations that induced both direct and indirect rearrangements (Figure 1 and Figure 4). This mutation type grouped most of mutations. Figure 4 showed that the L99F mutation produced large shitfs in the mutated residues and also in its neighbor residues 68 and 69.

The location of MCS atoms (Figure A3) in mutant structures highlighted structural rearrangments located in important regions for PR2: in the elbow and flap regions that are implied in the PR2 deformations induced by ligand binding, in its pocket binding and in its interface. In the following, we explored the impacts of the studied resistance mutation in the PI-binding pocket and PR2 interface.

### 2.2. Impact of Mutations on the Properties of PI-Binding Pocket

**Figure A4:**
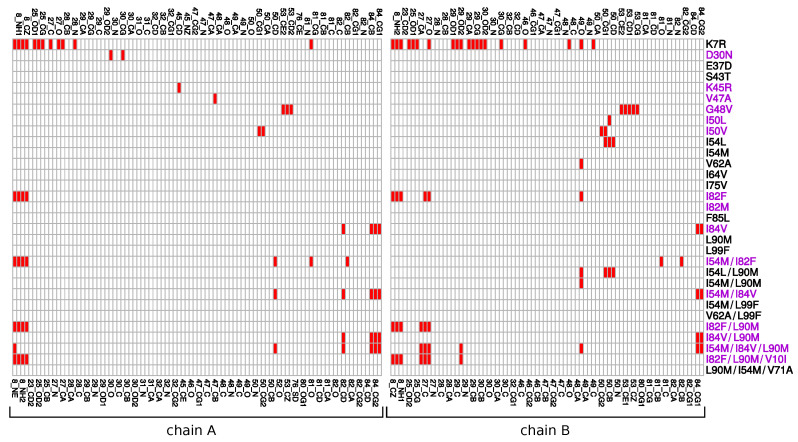
MCS atoms in the pocket. Mutants colored in purple present at least a mutation located in the binding pocket.

From the 31 mutants, 15 had at least one mutation in the binding pocket (Figure 1A and Figure A4). Except the I82M mutant, all these mutants presented MCS atoms in the pocket in the mutated or non mutated residue. Surprisingly, structural rearrangements in the binding pocket were also observed in five mutants without pocket mutations (K7R, I54L, V62A, I54L/L90M, I54M/L90M, Figure 1). A total of 36% of pocket atoms were deformed in at least one mutant, with an overrepresentation of side-chain atoms (Pearson’s Chi-squared Test *p*-value = $1\times 10^{-3}$), see Figure A4.

**Figure 5:**
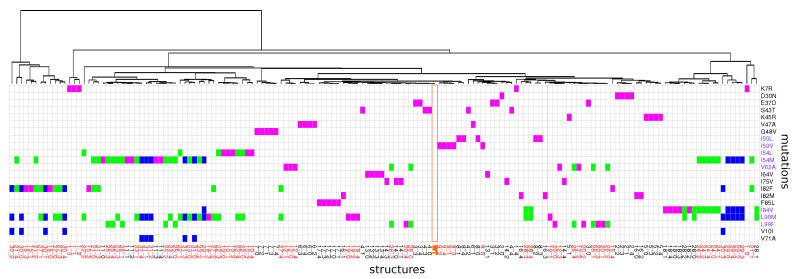
Hierarchical classification of wild-type and mutant pockets according to their conformational similarity quantified by pairwise RMSD computing using all pocket atoms. The table provides the description of each mutant structure in terms of mutations. Mutant structures are ranked according to their apparition in the classification. Single mutants are colored in magenta, double mutants in green and triple mutants in blue. The orange column locate the wild-type pocket. Mutations colored in purple correspond to mutations located in the binding pocket and mutant colored in red contain a MCS atom located in the binding pocket.

**Figure 6:**
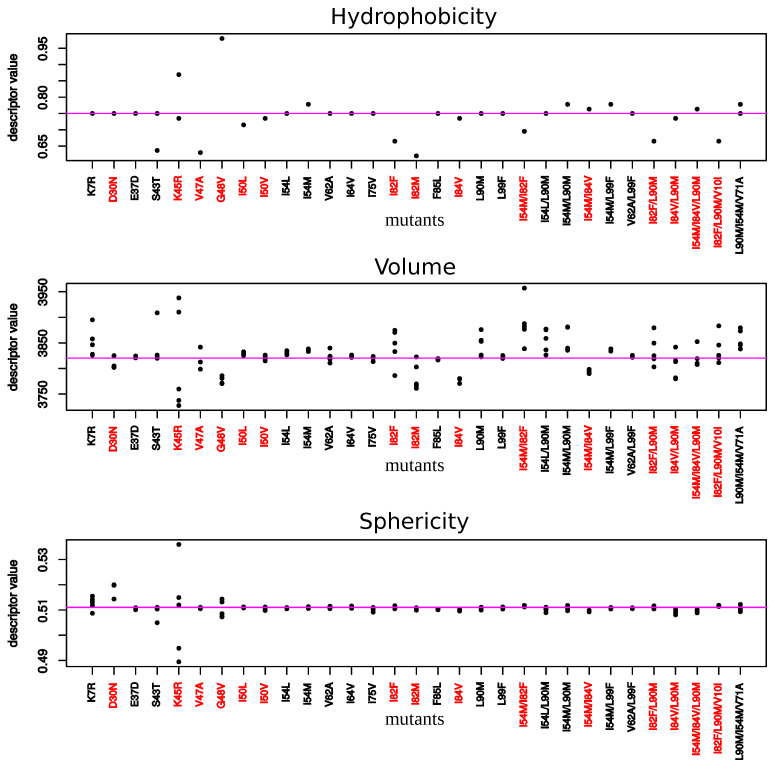
Hydrophobicity, volume, and sphericity of pockets extracted from each mutant structure. Red mutants correspond to mutants exhibiting at least one mutation in the binding. Each point corresponds to a mutant structure. For some mutants, less than five points appear meaning that several mutant structures exhibit same values for a given descriptor.

**Figure A5:**
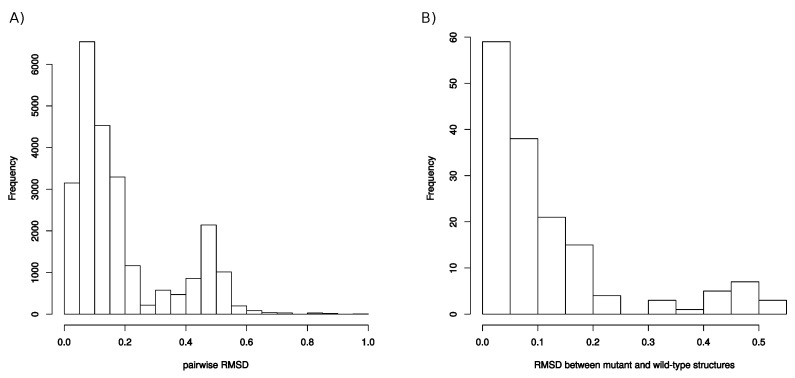
Comparison between pockets. (**A**) Distribution of RMSD computed between all pocket pairs. (**B**) Distribution of RMSD computed between the wild-type and mutant pockets.

To explore impacts of these mutations on the conformation and properties of the PI-binding pocket, PI-binding pockets were extracted from the 156 structures (1 wild-type and the 155 mutant structures). These 156 pockets were then classified according to their structural similarity quantified by pairwise RMSD (Figure A5 and Figure 5). In addition, their volume, sphericity, and hydrophobicity values were compared to those of the wild-type pocket (Figure 6). First, the five structures of a given mutant were not always bundled in the classification or presented some variability in terms of descriptor values. This highlighted a structural diversity of the five structures of mutants. This is explained by the minimization effects and the fact that several rotamers were possible for some amino acids during the mutagenesis process as illustrated by Figure A1 for the K45R mutation. Except structures of the I82M, I54M, L90M, I54M/L99F, and I54M/V71A/L90M mutants, most structures of mutants without MCS in pocket were close to the wild-type pocket in the hierarchical classification and presented similar descriptor values than the wild-type pocket (Figure 5 and Figure 6). Pocket of mutants with the K7R, I54M, I54L, I82F, and I84V mutations were the farthest to the wild-type pocket in the classification, meaning these mutations had the most impact on the pocket structure (Figure 5). Figure 6 showed that the K7R and I82F mutations also strongly modified pocket properties, like the K45R, V47A, G48V, I82M/F, and L90M mutations. More precisely, the V47A, I82F, and I82M mutations strongly decreased the pocket hydrophobicity. The I50V, I50L, V62A, and I84V mutations also caused a reduction of pocket hydrophobicity but with a weaker magnitude. The I50L and I82F mutations were also responsible of an increase of the pocket volume, in contrast to the I82M and I84V mutations that caused a reduction of the pocket volume. The G48V and I54M mutations increased the hydrophobicity of the pocket that was accompanied with a modification of the pocket size: the G48V mutation led to a reduction of the pocket volume in contrast to the I54M. An increase of pocket volume was also observed in the K7R, I54L, and L90M mutants with different magnitudes and a decrease of the pocket volume in the D30N and V47A mutants. The volume modification of the pocket of the K7R and D30N mutants was accompanied with an increase of the sphericity of the pocket. The K45R mutant was distinct because its five structure presented large diversity in terms of descriptor values Figure 6. Two of these pockets were bigger and less hydrophobic than the wild-type pocket while the three others were smaller, more hydrophobic and more spheric than the wild-type pocket.

Figure A4 presents the MCS atoms occurring in the binding pocket in each mutant. We noted that some mutations induce structural rearrangements in residues important for the PI binding. For example, the K7R, I50L/V and I54L mutations caused structural deformations in residues 25, 27, 30 that establish hydrogen bonds with PIs [18,33]. The D30N, K7R, I82F, I84V mutations led to atomic displacements in residues involved in van der Waals interactions with PI, such as in residues 23B, 27A, 28A, 30A, 49B, 48B, 82A and 84A.

Thus, the K7R, K45R, V47A, G48V, I50V/L, I54M, V62A, I82M/F, I84V, and L90M mutations could impact ligand binding by modifying pocket properties or the network of interactions with PIs.

### 2.3. Impact on Interface

**Figure 7:**
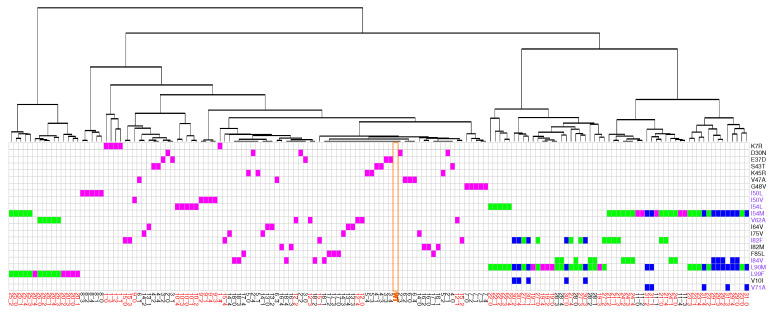
Classifications of the 156 structures according to their similitude of their interface. The table provides the description of each mutant structure in terms of mutations. Mutant structures are ranked according to their apparition in the classification. Single mutants are colored in magenta, double mutants in green and triple mutants in blue. The orange column locate the wild-type interface. Mutations colored in purple are invoved in the interface. Residues colored in red correspond to MCS atoms in the interface.

**Figure 8:**
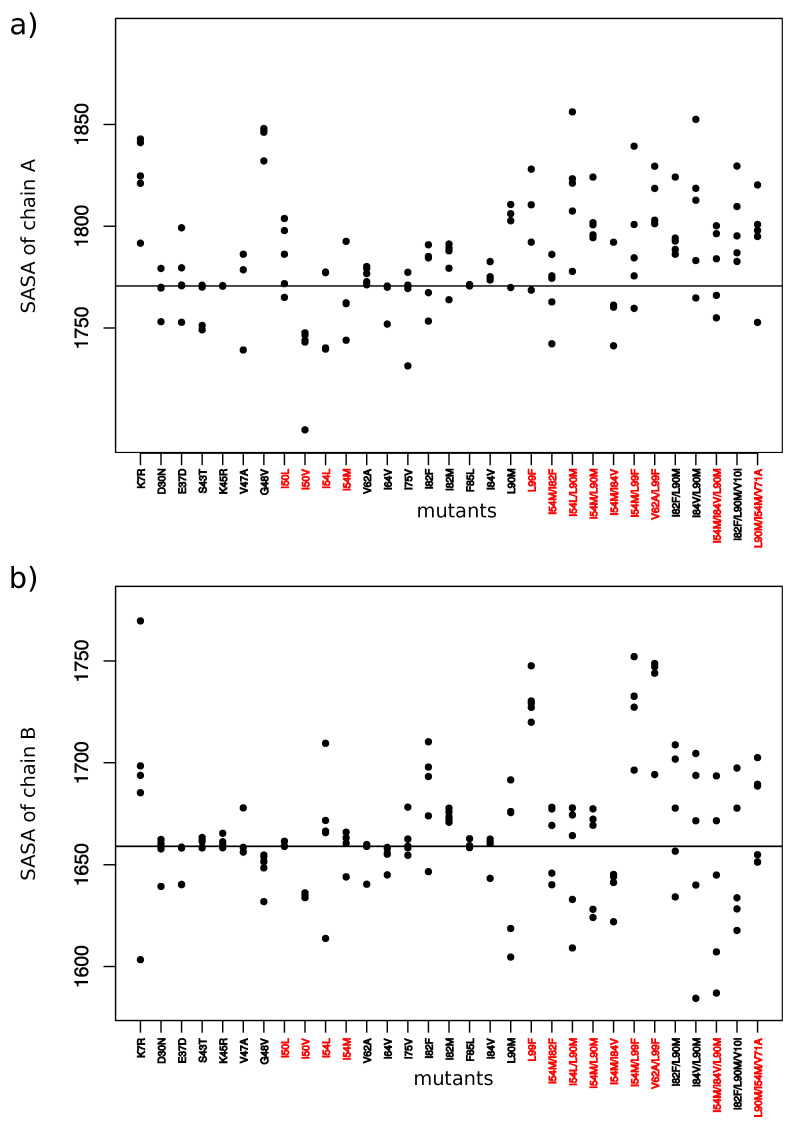
SASA values for interface of chain A (**a**) and chain B (**b**) for each mutant. Red mutants correspond to mutants having at least one mutation in PR2 interface.

**Figure A6:**
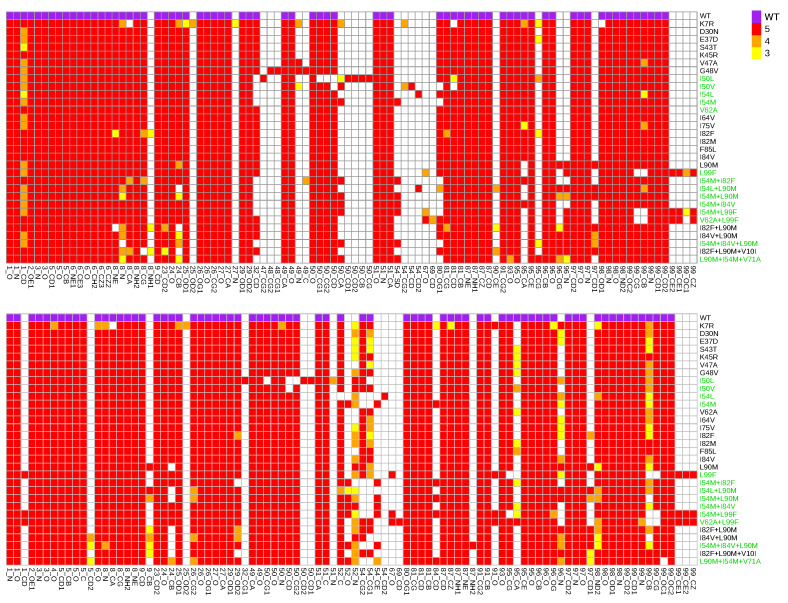
Number of mutant structure where a given atom is involved in the interface. Mutants colored in green present at least a mutation involved in the interface.

From the 31 drug-resistant mutants, 13 had at least one mutation in the PR2 interface (Figure 1A). Except the I50L and I54M mutants, all these mutants contained MCS atoms in their interface. Five mutants without mutation in the interface presented structural deformations in their interface. To analyze the impact of these mutations on the PR2 interface, interfaces of the wild-type and mutant structures were extracted and compared in terms of amino acid composition and their size. To do so, a hierarchical classification of the 156 interfaces was computed according to their similarity in terms of interface composition (Figure 7). In addition, the Solvent Accessible Surface Area (SASA) value, measuring the interface size, of the two parts of the interface was computed for each mutant structure using NACCESS software [37] (Figure 8). Figure 7 showed that most structures without MCS atoms in interface were close to the wild-type interface in the classification, revealing that these mutations led weak changes in the PR2 interface. This was confirmed by the fact that these interfaces had similar size than the wild-type interface (Figure 8). Three mutants (I50L, I54M and I84V/L90M) without MCS atom in interface exhibited different interface composition relative to the wild-type. These differences in terms of interface composition led to an increase of the size of chain A interface in the I50L and I84V/L90M mutants. The G48V mutation was responsible of the presence in the interface of the two side-chain atoms of residue 48_A and the absence in the interface of one atom of residue 95_B and 99_B relative to the wild-type interface (Figure A6), resulting that the chain A interface of the mutant was larger and this of chain B was smaller than the wild-type interface (Figure 7). These differences in terms of interface observed in the I50L, I54M, and G48V mutants were explained by supplementary atoms in their interface induced by the mutation (Figure A6).

From mutants having MCS atoms in the interface, the I50V, V62A, and I82F mutants corresponded to mutants inducing the less modifications in the PR2 interface (Figure 7 and Figure 8). This was expected for the V62A mutant because only one MCS atom was observed in its interface. It was more surprising for the I50V and I82F mutants because large deformations were detected in interface-residues 50 and 8 of chains A and B, respectively. In contrast, the K7R, I54L, L90M, and L99F mutations alone or in combination with others contained many MCS atoms in the interface that strongly modified its composition and its size. The K7R, L90M, and L99F mutations caused an increase of the size of the two parts of PR2 interface, and the mutation I54L induced a weak increase of the size of the interface of chain B.

### 2.4. Impact of Combining Several Mutations Relative to Single Mutant

Figure 3 showed that most multiple mutants contained many MCS atoms with large magnitude, i.e., with $dist^{WT-mutant}$ distance higher than 2 Å. However, combining several mutations did not significantly increase the average number of MCS atoms per mutant (Student’s *t*-test *p*-value = 0.59, Figure 3B). Comparison of MCS atoms in the single and multiple mutants showed that several multiple mutants exhibited specific MCS atoms relative to the corresponding single mutants. For example, we highlighted a displacement in atoms of residue 50_B in the I54L and I54L/L90M mutants but the shift was larger in the double ($dist^{WT-mutant}$ for CD_50_B and CG1_50_B atoms >2.5 Å) than in single mutants ($dist^{WT-mutant}$ for CD_50_B and CG1_50_B atoms was of 0.69 and 0.62 Å in the I54L mutant). Combining several mutations could induce apparition or loss of MCS atoms relative to the corresponding mutants. For example, the combination of the I54M and I82F mutations caused structural shifts in pocket residues 50_A, 81_A/B, and 82_A/B, while no shift at these residues were observed in the I54M and I82F mutants (Figure A4). In contrast, the I82F mutant contained a large shift at residue 8_B, a residue involved in the PR2 interface and pocket, those were not found in the I54M/I82F mutants (Figure A4). These structural changes in the double mutant relative to the I82F mutant induced a weak decrease of the interface size and an increase of the pocket volume (Figure 6).

### 2.5. Impact of Using Different Structure Modeling Software

**Figure 9:**
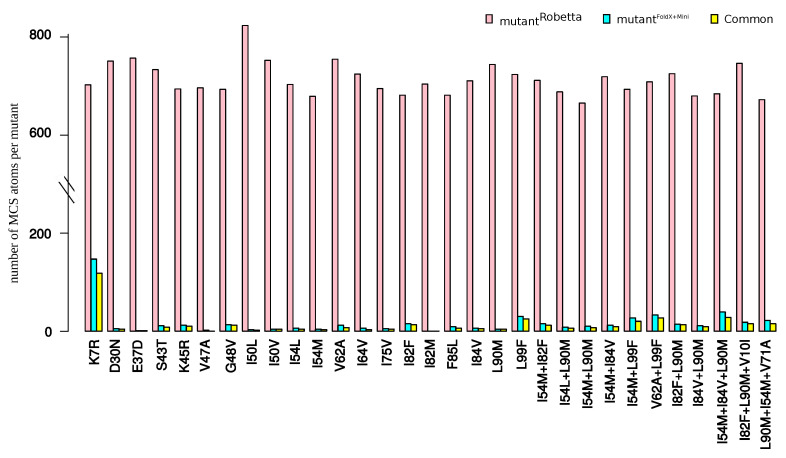
Number of MCS atoms in the $mutant^{Robetta}$ set, in the $mutant^{FoldX+mini}$ set, and observed both in the two mutant sets.

**Figure A7:**
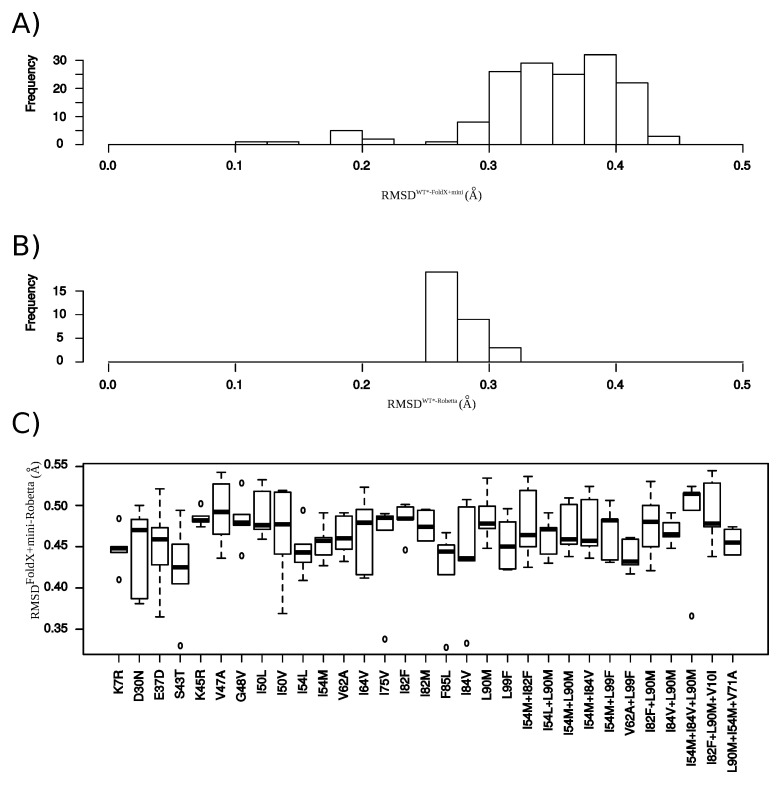
Comparison between $mutant^{FoldX+Mini}$ and $mutant^{Robetta}$ structures. (**A**) RMSD computed between the wild-type structure (not minimized, $WT^{*}$ structure) and $mutant^{FoldX+Mini}$ structure. (**B**) RMSD computed between the wild-type structure (not minimized, $WT^{*}$ structure) and $mutant^{Robetta}$ structure. (**C**) RMSD computed between each $mutant^{Robetta}$ structure and the five structure of $mutant^{FoldX+Mini}$.

**Figure A8:**
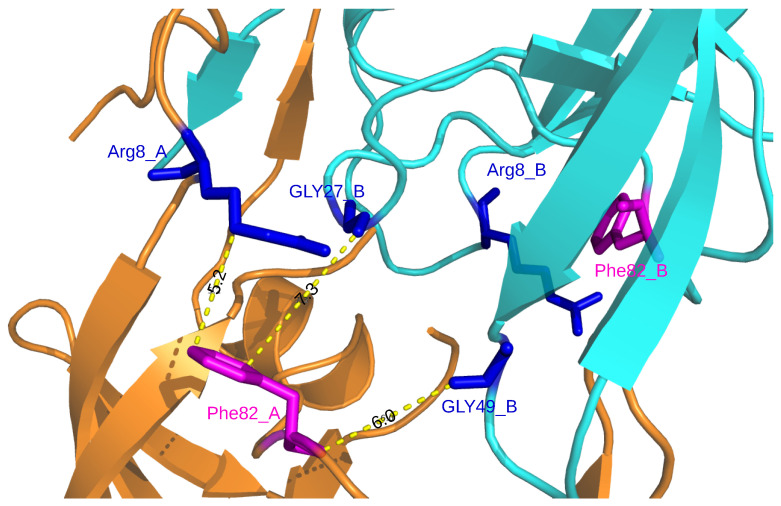
Location of residues exhibiting structural shifts in the I82F mutant. Chains A and B are presented in cartoon mode and colored in orange and blue, respectively. Mutated residues (82) are colored in magenta and presented in stick mode. Residues having shifted atoms are colored in blue and presented in stick mode.

In this section, we explored the impact of using another structure-modeling software in the detection of structural rearrangements induced by mutations. To do so, we modeled the structure of the 31 mutants using the webserver Robetta, see Appendix G. These mutant structures were denoted as $mutant^{Robetta}$. For a better clarity of this section, the mutant structures built using our initial protocol (based on FoldX software and an energetic minimization step) were denoted as $mutant^{FoldX+Mini}$. First, we compared mutant structures generated with the wild-type structure (crystallographic structure), Figure A7A,B. We noted that the protocol based on FoldX software plus a minimization step led to a set of structures exhibiting a larger diversity than Robetta webserver. Then, the two mutant sets were compared by computing RMSD between $mutant^{Robetta}$ structures and the five structures of $mutant^{FoldX+Mini}$, denoted as $RMSD^{WT^{*}-mutant^{Robetta}}$. The two modeling protocols led to different structures with an average RMSD of 0.46 ± 0.04 between $mutant^{Robetta}$ and $mutant^{FoldX+Mini}$ structures (Figure A7C). We then compared the number of MCS atoms oberved in each structure of the two mutant sets, see Appendix G.3. Figure 9 presents the number of MCS atoms detected in each mutant. We noted that $mutant^{Robetta}$ structures exhibited substantially more MCS atoms than $mutant^{FoldX-mini}$ structures. This was explained by two reasons. First, the determination of the MCS atoms in $mutant^{FoldX-mini}$ structures was based on the comparison of the mutant structures and the minimized wild-type structures, while MCS atoms in $mutant^{Robetta}$ structures were detected by comparing mutant structures with the non minimized wild-type structure. The second reason was that an atom was detected as a MCS atom in the $mutant^{FoldX-mini}$ if it had a $dist^{WT-mutant}$ larger than 0.3 Å in at least three structures of the mutant. Figure 9 presents the number of atoms detected as MCS in both mutant sets. From the 504 MCS atoms per mutants detected in the $mutant^{FoldX+mini}$ set, 78% were detected as MCS atoms in the $mutant^{Robetta}$ set. Although, the protocol based on FoldX software minimized the detection of structural rearrangements, the extracted structural deformations using this protocol was mainly found by a protocol based on another modeling software.

## 3. Discussion

In this paper, we proposed a quantification and location of structural deformations at backbone and side-chain atoms of PR2 occurred in an update set of 31 drug-resistant mutants based on their modeled structures. We detected a set of atoms presenting a shift in at least one mutant structure relative to the wild-type structure. To identify structural rearrangements resulted from the mutation, we then retained only MCS atoms, i.e., atoms with a distance between its position in the mutant and wild-type structure higher than 0.3 Å in at least three of structures of a given mutant. The distance cutoff was set up to 0.3 Å to select only significant shifts and considering uncertainties in the X-ray structure, like in Liu et al. [36]. This step allowed us to detect on average 16.26 ± 26.09 MCS atoms per mutants. However, these two cutoffs could led to an under-estimation of detected structural shifts. This could explain the fact that no MCS atom was detected in the I82M mutant, while the binding pocket extracted from this mutant was smaller and less hydrophobic than the wild-type pocket. Indeed, using a distance cutoff of 0.1 Å, we counted 96 ± 107 MCS atoms per mutants and 17 MCS atoms in the I82M mutant.

The analysis of MCS atoms showed that they occurred in side-chain and backbone atoms in the mutated residues, in its vicinity or further in the structure. We noted that structural rearrangements in side-chain was more frequent and with larger magnitude than those observed in backbone atoms. Thus, drug-resistance mutations induced more deformations in side-chains than in backbone atoms. These results suggest that to combat against HIV drug resistance, it would be interesting to develop inhibitors that establish hydrogen interactions with backbone atoms. Favor backbone interactions between PI and PR is a strategy used for the design of DRV to avoid the detrimental effects of resistance mutations [38,39].

Our results showed that the studied drug-resistance mutations impacted all PR2 regions. However, even though mutations occurred in the two PR2 chains, we noted an assymetry in the impact of mutations in the two chains. This assymetric behavior of mutations is linked to the fact that PR2 is a structural asymmetric protein, i.e., its two chains exhibit different conformations in unbound and bound forms [11,12,35,40,41,42,43]. This structural asymmetry of PR2 could result from crystal packing, ligand binding, and intrinsic flexibility of PR2 [11], and may be involved in the structural changes of PR2, particularly upon ligand recognition and binding [11,12,41,42]. Our results are in agreement with previous findings that have showed that drug-resistance mutations could modify PR2 structural asymmetry [31]. Thus, as PR2 is an asymmetric protein, resistance mutations do not always have the same impact on the two chains.

The location onto PR2 structure of MCS atoms highlighted structural deformations that could be linked to resistance mechanisms. First, we observed, in 19 mutant structures, structural deformations in binding pocket residues as reported in Laville et al., 2020 [31]. Most of these mutations were located in the binding pocket, except the K7R, I54M and L90M mutations. Our findings revealed that the S43T, V47A, K45R, G48V, I82F, and I82M strongly modified pocket hydrophobicity. The two first decreased the hydrophobicity, while the four last increased it. The I50V, I50L, V62A, and I84V mutations induced also a modification of the pocket hydrophobicity but with a very weak magnitude. The K7R, I50L, I54M, I54L, I82F, and L90M mutations increased the pocket volume, while the D30N, V47A, G48V, I82M, and I84V mutations had the opposite effect. By comparing PR1 and PR2 binding pocket, we have previously observed that amino-acid changes occurring in pocket residues 31, 32, 46, 47, 76, and 82 increased the hydrophobicity of the binding pocket [19]. Chen et al. (2014) reported that these mutations have also an impact on the volume of the binding pocket [22]. In addition, the K7R, D30N, I50L/V, I54L, I82F, and I84V mutations seemed to have direct impact on the PI binding by causing structural rearrangements in pocket residues that establish hydrogen and van der Waals interactions with PIs. Secondly, our finding reported that mutations K7R, E37D, S43T, K45R, V47A, G48V, I50V, I50L, I54M, I54L, and V62A, occurring alone or in combination with others, induced directly or indirectly structural changes in the elbow and flap regions. These two regions are known to be important in the opening and closing mechanisms of the binding pocket during ligand binding [10]. Thus, these mutations could impact PI binding by modifying the flexibility and movement of the flap region upon PI binding. Thirdly, we reported that the K7R, I50L, I54L, G48V, L90M, and L99F mutations caused structural displacements that impacted the composition and size of the PR2 interface. This suggested that these mutations may alter the stability of PR2.

Several drug-resistant mutations were structurally studied in PR1 by comparing crystallographic structures of the wild-type and mutants in bound and unbound forms. These studies showed that drug-resistance mutations alter the conformation of flap residues and flap dynamics, modifying binding pocket properties and the interaction network with PI, and the PR2 stability. For example, it has been shown that some resistance mutations, such as the M46L, G48V, I50V, and I54V/M mutations, alter the conformation of flap residues and flap dynamics in PR1 [36,44,45,46,47,48]. The impact of resistance mutations on flap conformation had also observed in several multi-drug resistant mutants such as the PR20 (including 20 mutations), Flap+ (with L10I, G48V, I54V, and V82A mutations), and MDR-769 (with nine mutations) mutants having more opened flap region relative to the wild-type PR1 [49,50,51,52]. In addition, Shen et al., (2015) suggested that the E35D, M36I, and S37D mutations in the multi-drug resistant PRS17 mutant induce an increase of the flexibility of the flap region [53]. The impact of the amino-acid changes at residues 30, 50, 82, 84, and 90 on the pocket volume have been also observed in PR1 [48,49,52,54,55,56,57]. For example, the V82A and I84V mutations lead to an expansion of the active-site cavity [52,54], while the V82F and L90M mutations cause a volume reduction in the binding cavity [48,49,54,57]. The direct impact of mutations occurred in residues 30, 50, 82, and 84 on the network of PR-PI interactions were previously observed in PR1 [51,57,58,59,60,61,62]. The impact of the L90M mutation on the PR2 stability was previously observed in PR1 using urea denaturation experiment [63,64] or using sedimentation equilibrium analysis [65]. However, we noted several disagreements between findings obtained in PR1 and PR2. For example, Liu et al., (2008) observed structural deformations around the tip of the flap and 80 s loop (residues 78–82) in PR1 mutants G48V, I50V, I54V/M complexed with SVQ and DRV [36]. Our analysis of PR2 mutants detected displacements of atoms located around the flap tip in the I50V, I54M, and G48V mutants and in residue 53 in the G48V mutant. However, structural deformations in the 80 s loop is only found in the I54M mutant with a weak shift in backbone atom 79_B_0 ($dist^{WT-mutant}$ of 0.44 Å). An important structural rearrangement of the main-chain of residue 25 in the PR1 mutant L90M linking to the L90M resistance was observed in several studies [49,57]. Our result did not highlight structural deformations at atoms of residue 25 in the L90M mutant of PR2; atoms of residue 25A/B exhibit an average $dist^{WT-mutant}$ of 0.07 ± 0.02 Å. In addition, we noted that the I84V and L90M mutations have not the same effect on the binding pocket in PR1 and PR2. Indeed, our findings showed that, in PR2, the I84V led to a reduction of pocket volume and the L90M mutation induced an augmentation of the pocket size in contrast in PR1 [48,49,52,54,57]. These disagreements in terms of impacts of drug-resistance mutations in PR1 and PR2 could be explained by the methodological differences of the two approaches. Indeed, our study was based on modeled structures, while PR1 studies used crystallographic structures. However, another reason that could explained these disagreements is the structural differences between PR1 and PR2 structures [13,18,19,40,42] leading to different PI-resistance profiles: PR2 is naturally resistant to six of the nine FDA (Food and Drug Administration)-approved PIs available for HIV-1 therapy [1,3,4]. More particularly, our previous work showed that the α-helix region (87–95), containing residue 90, presents different conformations in PR1 and PR2 and these structural differences could be partially explained by amino acid substitutions observed between the two PRs [13]. In addition, we previously showed that two PRs exhibit pockets with different properties: PR2 pockets are smaller and more hydrophobic than PR1 pockets [19,22]. These observation suggested that a same mutation could have different impacts on PR1 and PR2 structures.

In this study, we explored the impact of drug-resistance mutations on PR2 structure. To do so, we modeled 3D structures of mutant using as template the PR2 complexed with DRV (PDB code: 3EBZ [33]). However, it has been shown that drug-resistance mutations exhibit different sensitivities to the nine FDA-approved drugs [2,4,8,10,26]. For example, the I54M mutation lead to moderate resistance for indinavir, nelfinavir, tripanavir, DRV, and LPV, a high resistance for amprenavir, and is susceptible to SQV [26]. Thus, it would be interesting to consider different PIs and to cross information about the complete resistance profile of each mutant and the detected structural deformations. However, reliable resistance profile for PIs are difficult to collect for all mutants as few studies are available [3,4,26,29] and some of these studies have led to opposing results [10].

## 4. Materials and Methods

### 4.1. Data

In this study, we started from the list of the 30 drug-resistant mutants of PR2 studied in our previous study [31]. This mutant list was updated and a list of 31 drug-resistant mutants of PR2 was selected. This mutant set contains 22 different mutations (Figure 1) and there are 20 single mutants (with one mutation), 8 double mutants (with two mutations), and 3 triple mutants (with three mutations). These mutations sample the entire PR2 (Figure 1).

### 4.2. Structure Modeling

We modeled the 3D structure of each mutant using the protocol used in Laville et al., 2020 [31] based on FoldX suite [32] and Gromacs software [66]. This two-step protocol was applied to the wild-type crystallographic structure of the PR2 complexed with DRV (PDB code: 3EBZ [33]). First, the DRV ligand, metal atoms, and water molecules were removed from the crystallographic structure of PR2. Then, the RepairPDB command of the FoldX suite [32] was applied to the PR2 structure to reduce its energy. The in silico mutagenesis was then performed using the BuildModel command of the FoldX suite based on a side-chain rotamer library [32]. This step was performed five times to consider the several rotamers available for each amino acid [32] and generated five structures per mutant. An energetic minimization was then applied to the five modeled structures of a mutant using the protocol developed in our previous study [31]. We applied PROPKA software [67] to monoprotonate the oxygen atom OD2 of Aspartate 25 in chain B. Then, the system was solvated in a truncated octahedron box of explicit solvent (TIP3P water model) with a 12.0 Å buffer in each dimension and its charge was neutralized using chloride ions. The minimization of the system was performed using a two-step protocol using the force field AMBER ff99SB in GROMACS [66] by applying a steepest descent algorithm combined with a conjugate gradient algorithm. A first step energy minimization allowed relaxing water molecules and counterions using a position harmonic restraining force of 100 kcal·mol${}^{-1}$Å${}^{-2}$ on the heavy atoms of the protein. Then, restraints on protein atoms were removed using a second energy minimization step. The protocol used the particle mesh Ewald (PME) method to treat the long-range electrostatic interactions [68] and a cutoff distances of 10.0 Å for the long-range electrostatic and van der Waals interactions. This protocol was applied to the 31 mutants generating 155 mutant structures (5 structures per mutant). The minimization protocol was also applied to the wild-type structure and the minimized wild-type structure was named wild-type structure.

### 4.3. Identification of Shifted Atoms Induced by Mutations

First, all shifted atoms in each mutant structure were extracted by comparing the position of atoms in the wild-type and mutant structures. To do so, we applied the method used in Perrier et al., 2019 [69]. Each mutant structure was superimposed onto the wild-type structure (the minimized wild-type structure) using PyMOL software [70]. Superimposition was based on all atoms. Hydrogen atoms were removed. Euclidean distances between the position of each atom in the mutant and wild-type structure were computed. These distances were denoted as $dist^{WT-mutant}$. Higher a $dist^{WT-mutant}$ value of an atom is, more the atom is shifted in the mutated structure relative to the wild-type structure. According to the modelization protocol, detected atom shifts resulted from the mutagenesis and minimization. To retain only significant structural rearrangements induced by resistance mutation, only atoms with a $dist^{WT-mutant}$ value higher than 0.3 Å were retained like in Liu et al., 2008 [36]. This distance cutoff of 0.3Å allowed selecting only significant structural displacements and removing uncertainties in the X-ray and mutant structures [36]. In addition, for each mutant, only shifted atoms observed in three of the five structures of the mutant were retained. These shifted atoms were named mutant-conserved shifted and noted MCS atoms.

### 4.4. Comparison of Wild-Type and Mutant Pockets

#### 4.4.1. Pocket Estimation

From the 156 structures, we extracted the binding pocket. To consider the fact that the different known PI are structurally diverse and that resistance mutations induce resistance to one or several PI, we used the “common-ligand” approach to estimate pockets [11] that consisted to define the binding pocket as all atoms of PR2 capable to bind all co-crystallized ligands. To do so, a virtual ligand was built by superimposed all co-crystallized ligands extracted from a set of PRs. This virtual ligand was then placed in the query structure and its pocket was estimated as atoms located at least 4.5 Å of the virtual ligand. This protocol was applied on the 156 wild-type and mutant structures using the virtual ligand built from the PR set used in Triki et al., 2018 [11]. The pocket extracted from the wild-type structure was denoted as wild-type and those extracted from mutant structures were denoted as mutant pockets.

#### 4.4.2. Comparison of the Conformation of Wild-Type and Mutant Pockets

To compare the conformation of the 156 pockets, we computed the root mean square deviation (RMSD) between each pocket pair (156×156). The calculation of RMSD was performed using PyMOL software [70] based on all pocket atoms. From these RMSD values, we computed a hierarchical classification of the pockets using the Ward method aggregation.

#### 4.4.3. Comparison of the Properties of Wild-Type and Mutant Pockets

Each pocket was characterized by two geometrical descriptors (VOLUME_HULL and PSI), and one physicochemical descriptor (hydrophobicity_kyte) [71]. The VOLUME_HULL descriptor provides an estimation of the volume of a pocket. PSI measures the sphericity of a pocket. These two descriptors were computed using PCI software [72]. hydrophobicity_kyte descriptor quantifies the hydrophobicity of a pocket.

### 4.5. Interface Comparison

#### 4.5.1. Interface Extraction

The PPIC (Protein-Protein Interface Computation) program [73,74] was used to determine atoms involved in the interface of a structure. This program is parameter-free. It takes in input the 3D structure of a complex with two molecules (molecules or macromolecules) A and B. It defines the interface between the two molecules in two parts: interface of A and interface of B. Each interface part corresponds to the non-redundant set of all nearest neighbor atoms in one molecule of the atoms of other molecules. The extraction of neighbor atoms is performed using a simpler method of the Voronoï tessellation method [75]. Contrary to the Voronoï tessellation method, PPIC approach does not generate neighbors at long distances in the interface.

We used PPIC program to extract interface from the wild-type structure, denoted as wild-type interface, and the interface from the mutant structures, denoted as mutant interface.

#### 4.5.2. Comparison of the Interface Composition

We compared the composition of the 156 interfaces by computed the Hamming distance, noted $D_{interface}$ between each interface pairs (Equation (1)).

(1) $$D_{interface}\left(I1,I2\right)=N_{I1}+N_{I2}-2\times N_{I12}$$

with I1 and I2 the interfaces extracted from two structures, $N_{I1}$ and $N_{I2}$ the number of atoms of the interfaces I1 and I2, respectively, and $N_{I12}$ the number of common atoms of the two interfaces I1 and I2.

Higher $D_{interface}\left(I1,I2\right)$ is, more dissimilar the two interfaces I1 and I2 are. To facilitate the interpretation of these distances, each $D_{interface}\left(I1,I2\right)$ value was normalized by the maximum number of atoms in the two interfaces ($N_{I1}+N_{I2}$) using Equation (2), resulting in the computation of the $D_{interface}{\left(I1,I2\right)}^{norm}$ value for each interface pair.

(2) $$D_{interface}{\left(I1,I2\right)}^{norm}=\frac{D_{interface}\left(I1,I2\right)}{N_{I1}+N_{I2}}.$$

The $D_{interface}{\left(I1,I2\right)}^{norm}$ is ranking from 0 to 1, with a $D_{interface}{\left(I1,I2\right)}^{norm}$ equal to 0 means that the composition of the two interfaces I1 and I2 is identical.

Using the $D_{interface}{\left(I1,I2\right)}^{norm}$ values of each interface pairs, we computed a hierarchical classification of the 156 structures allowing to group structures according to their similarity in terms of interface composition. This classification was computed using the Ward method aggregation.

#### 4.5.3. Comparison of the SASA of Interface

The SASA of the interface of each structure was determined using NACCESS software [37]. First the two monomers of each structure were separated. Then, the accessible surface area of all atoms of the 312 monomers was computed using NACCESS software [37]. The SASA values of the two parts of the interface (chains A and B) of a structure were obtained by computed the sum of the SASA of each atom detected as involved in the interface. The SASA value reflects the size of a interface.

## 5. Conclusions

In this study, we explored the impact of drug-resistance mutations reported in PR2. We first compared the modeled structure of 31 mutants with the wild-type PR2 structure to locate structural rearrangements induced by mutations. Secondly, we studied the impact of these deformations on the conformation and properties of the binding pocket and on the interface. Our findings showed that one mutation could induce large structural deformations located in the mutated residue, in its vicinity or far in the structure. These structural deformations occur in both side-chain and backbone atoms, with on average more impact in the former. However, we revealed that resistance mutations do not always have the same behavior in the two monomers of PR2, which is link the structural asymmetry of this target.

The analysis of the location of structural rearrangements induced by resistance mutations provided clues to better understand resistance mechanisms. First, some mutations have a direct or indirect impacts on PI-binding. The K7R, V47A, G48V, I50L/V, I54L/M, V62A, I82F/M, I82M, I84V, and L90M induce structural rearrangements in the binding pocket that modify its conformation, volume and/or hydrophobicity. These changes in the binding pocket could have a negative effect on PI binding. In addition, some of these mutations (K7R, D30N, I50L/V, I54L, I82F, and I84V) have a direct impact on PI binding by causing structural displacements of residues establishing interactions with PI. Resistance mutations have also an impact on conformation of the elbow and flap regions, regions involving in the transition of the open and closed conformations of the PR2 upon ligand binding. Indeed, we reported that the K7R, E37D, S43T, K45R, V47A, G48V, I50V, I50L, I54M, I54L, and V62A led atom shifts in the elbow and flap regions that could modify the flexibility and movement of the flap region important in the binding-pocket closing. Thirdly, the K7R, G48V, I50L, I54L, L90M, and L99F mutations induce structural rearrangements in the PR2 interface that modify its composition and size that may alter the stability of PR2. Finally, we highlighted several residues that were never deformed in mutant structures.

In conclusion, this study explored the impact of a large number of PR2 resistance mutations on PR2 structure, particularly on its pocket binding and interface. Our results showed that some structural rearrangements induced by resistance mutations are located in important regions of PR2: the elbow and flap regions inducing in the PR2 deformation upon ligand binding, in the PI-binding pocket and in the interface of the two monomers. Some of these deformations modify the properties of binding pocket and the composition and size of the PR2 interface. These results suggest that the studied resistance mutations could alter PI binding by modifying the properties and flexibility of the pocket or the interaction between PR2 and PI and/or alter PR2 stability. Our results reinforce the resistance mechanisms proposed in our previous study [31] and lead to a better understanding of the effects of mutations that occurred in PR2 and the different mechanisms of PR2 resistance.

## Data Availability

The data presented in this study are openly available in https://figshare.com/articles/dataset/Data_of_Laville_et_al_2021/13634147.

## Abbreviations

The following abbreviations are used in this manuscript:

PR	Protease
PR2	HIV-2 Protease
PI	Protease inhibitor
MCS atoms	Mutant-Conserved Shifted atoms
RMSD	Root Mean Square Deviation
DRV	Darunavir
LPV	Lopinavir
SQV	Saquinavir
IDV	Indinavir
3D	Three-dimensional
SASA	Solvent Accessible Surface Area
PPIC	Protein-Protein Interface Computation

## Appendix G

In this section, mutant structures built using our initial protocol (based on FoldX software and an energetic minimization step) were denoted as $mutant^{FoldX+Mini}$. The $WT^{*}$ abbreviation described the crystallographic structure of PR2 complexed to the DRV that corresponded to the wild-type structure that was not minimized.

### Appendix G.1. Protocol to Model Mutant Structures Using Robetta Software

Robetta webserver (https://robetta.bakerlab.org/) is a protein structure prediction service based on the RosettaCM method [76]. RosettaCM is a comparative modeling method that assembles structures using integrated torsion space-based and Cartesian space template fragment recombination, loop closure by iterative fragment assembly and Cartesian space minimization, and high-resolution refinement [76].

As in our initial modeling protocol, we used the PDB structure 3EBZ (PR2 complexed with DRV) as template. First, the DRV ligand, metal ions and water molecules were removed from the structure. Using this template, the structure of the 31 drug-resistant mutants was built using Robetta webserver with the “CM” option. Other parameters were set to defaults. This step resulted in a set of 31 mutant structures, named $mutant^{Robetta}$. The RMSD (based on all atoms) between the wild-type and $mutant^{Robetta}$ structures were computed using PyMoL software [70]. These RMSD values were refeered as $RMSD^{WT^{*}-mutant^{Robetta}}$.

### Appendix G.2. Structural Comparison between the $Mutant^{FoldX+Mini}$ and $Mutant^{Robetta}$ Structures

First, we compared the two sets of PR2 mutants. For each mutant, its $mutant^{Robetta}$ structure was superimposed onto the five $mutant^{FoldX+Mini}$ mutants and the RMSD based on all atoms was computed using PyMoL software [70]. These RMSD were denoted as $RMSD^{FoldX+Mini-Robetta}$.

### Appendix G.3. Detection of Structural Rearrangements in the Set of $Mutant^{Robetta}$ Structures

First, each $mutant^{Robetta}$ structure were superimposed onto the 3EBZ (PDB code) structure using PyMoL software [70]. Superimposition was based on all atoms. Euclidean distances between the position of each atom in the mutant and 3EBZ structure were computed. These distances were denoted as $dist^{WT^{*}-mutant^{Robetta}}$. An atom was considered as a MCS atom in a $mutant^{Robetta}$ structure if it had a $dist^{WT^{*}-mutant^{Robetta}}$ value higher than 0.3 Å.
